# Mid-Latency Auditory Evoked Potentials Differentially Predict Sedation and Drug Level Under Opioid and Hypnotic Agents

**DOI:** 10.3389/fphar.2018.01427

**Published:** 2018-12-04

**Authors:** Gernot G. Supp, Focko L. Higgen, Joerg F. Hipp, Andreas K. Engel, Markus Siegel

**Affiliations:** ^1^Department of Neurophysiology and Pathophysiology, University Medical Center Hamburg-Eppendorf, Hamburg, Germany; ^2^Department of Neurology, University Medical Center Hamburg-Eppendorf, Hamburg, Germany; ^3^Centre for Integrative Neuroscience – MEG Center, University of Tübingen, Tübingen, Germany

**Keywords:** EEG, AEP, anesthesia, opioid, propofol, remifentanil

## Abstract

**Background:** Auditory-evoked brain potentials (AEPs) are widely used to assess depth of the sedative component of general anesthesia. Depth of sedation as induced by hypnotic drugs (e.g., propofol) is characterized by a gradual decline of mid-latency cortical AEPs (10–50 ms). Using the decline of mid-latency AEPs as a reliable index for sedation requires its robustness against confounding pharmaceutical influences, e.g., analgesic opioids such as remifentanil. Critically, in this context the following two questions remained unresolved so far: First, it is unclear whether opioids directly affect mid-latency AEPs. Second, high doses of opioids decrease arousal, but it is unknown whether opioid-induced sedation is reflected by the diminution of mid-latency AEPs. We hypothesized that opioids affect mid-latency AEPs and that these effects rely on different mechanisms compared to hypnotic agents.

**Methods:** To address both questions, we performed a series of experiments under the participation of healthy human volunteers. We measured AEPs and quantified participants’ sedation state by a standardized rating scale during stepwise increase of different pharmaceutical agents (remifentanil, propofol or placebo).

**Results:** Our results revealed a decline of mid-latency AEPs during remifentanil medication. This decrease was predicted by drug dose, rather than sedation level. In contrast, attenuation of the mid-latency AEPs during propofol was predicted by sedation level and was not related to hypnotic drug dose. We did not find any drug-induced changes of brainstem AEPs (1–10 ms).

**Conclusion:** As remifentanil reduced mid-latency AEPs without inducing strong sedation levels, a decrease of this evoked brain component does not constitute an unequivocal index for the depth of sedation. These results challenge the use of mid-latency AEPs as a reliable marker of depth of the sedative component of anesthesia if hypnotic drugs are combined with opioids.

## Introduction

Worldwide, every year, more than 230 million patients are estimated to undergo anesthesia in the course of major surgical interventions (Weiser et al., 2008). To safely induce and maintain general anesthesia before and during surgery it is essential to monitor the patient’s actual state of vigilance, i.e., anesthesia’s sedative or hypnotic component (Brown et al., 2010). Auditory evoked brain responses are widely used in clinical practice to assess the depth of sedation (sedation level) during general anesthesia (Sebel et al., 1985; Davies et al., 1996; Mantzaridis and Kenny, 1997; Gajraj et al., 1998; Drummond, 2000; Loveman et al., 2001; Trillo-Urrutia et al., 2003; Bruhn et al., 2006; Haenggi et al., 2009; Stoppe et al., 2012).

Auditory evoked potentials (AEPs) can be separated on the basis of their latencies in three response components that reflect different cerebral stages of processing: brainstem AEPs (BAEPs, 1–10 ms latency), early or mid-latency AEPs (10–50 ms latency) and late cortical AEPs (50–500 ms) (Schwender et al., 1995; Plourde, 2006). Especially mid-latency AEPs are affected by hypnotic agents. In particular, drugs such as propofol, isoflurane, halothane, enflurane, and etomidate decrease mid-latency AEPs amplitudes in a dose-dependent, but agent-independent manner (Thornton et al., 1984, 1985, 1989, 1992; Heneghan et al., 1987; Chassard et al., 1989; Tooley et al., 1996; Palm et al., 2001; Kuhnle et al., 2013; Shi et al., 2014). As the level of sedation also increases with drug dosage, the decline of mid-latency AEPs is usually correlated with sedative depth. In contrast, brainstem components seem to remain unchanged during anesthesia (Thornton and Sharpe, 1998; Banoub et al., 2003). Late cortical AEPs are modulated by sedative depth, but also by other factors such as cognitive processes (Hillyard et al., 1973; Picton et al., 1974).

The validity to use the decline of mid-latency AEPs as an index for progressive sedation critically depends on its robustness against confounding influences of other pharmaceutical agents administered in combination with the hypnotic drug during general anesthesia such as, e.g., the analgesic opioid remifentanil (Schmidt et al., 2007; Fodale et al., 2008; Kortelainen et al., 2011). But so far, clinical studies have not provided a consistent answer as to whether or not opioids directly affect mid-latency AEPs. While Crabb et al. (1996) showed a modulating effect of remifentanil on mid-latency AEP component *Pa*, other studies did not find significant AEP changes due to opioid administration (Schwender et al., 1993, 1995; Iselin-Chaves et al., 2000; Tooley et al., 2004; Wright et al., 2004; Schraag et al., 2006).

Wright et al. (2004) argue that their conclusion that opioids do not depress mid-latency AEPs might be due to an insufficient dosage of remifentanil in their design and therefore point out the possibility of a direct dose-related effect. In addition, most studies showing no effect used a combined administration of opioid and hypnotic agents, which may render it more difficult to detect an effect of opioids.

Furthermore, high doses of opioids are known to affect the sedation level, i.e., they decrease the subjects’ vigilance (Stein et al., 2003; Schmidt et al., 2007). Therefore, it may be hypothesized that – analogous to the effect of hypnotic drugs – this opioid-induced sedation could be reflected in a decline of mid-latency AEPs.

In sum, two basic questions remain unresolved. First, do opioids such as remifentanil have a direct effect on mid-latency AEPs? Second, is such a potential effect related to a change of sedation level in ways similar to various hypnotic agents? High doses of opioids decrease arousal, but it is unknown whether opioid-induced sedation is reflected by the diminution of mid-latency AEPs.

To address both questions, we conducted a series of experiments under the participation of healthy human volunteers. We measured auditory stimulus-related electroencephalographic (EEG) activity during a step-wise increase of different pharmaceutical agents, either using the opioid remifentanil, the hypnotic drug propofol or placebo administration. In contrast to clinical settings, where propofol and remifentanil are frequently co-administered, the current experimental setup realized a separate administration of each drug. This setting allowed us to investigate the specific effects of each single pharmaceutical agent on the cortical brain activity in humans. We hypothesized that opioids affect mid-latency AEPs and that these effects rely on different mechanisms compared to hypnotic agents.

## Materials and Methods

### Participants

After fully informed written consent a total of nine (*n* = 9) healthy male adult volunteers (age range: 22–34 years; weight range: 69–100 kg) participated in the study, which was conducted in accordance with the Declaration of Helsinki and was approved by the local institutional review board (Ethik-Kommission, Ärztekammer Hamburg, Germany). Each participant received monetary compensation for his participation. All participants had no history of neurological disorders and no history of centrally acting drug intake.

### Study Design

Each participant underwent measurements on three different days, with an intersession interval of 1 week. During each session, participants received placebo or a single pharmaceutical agent, using either the opioid remifentanil or the hypnotic agent propofol in a random double-blind crossover design. To realize the double-blind setting, we used a concealed intravenous catheter, a black intravenous line and a syringe pump that was placed outside the investigation room.

**FIGURE 1:**
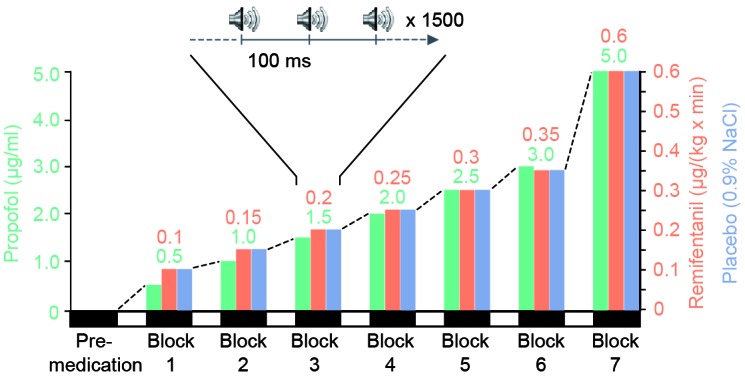
Experimental design. During three separate sessions, each participant (*n* = 9) received stepwise increasing medication of a potent short-acting opioid (remifentanil), a short-acting hypnotic drug (propofol) or placebo. For each session one block of data was recorded before drug-application (pre-medication). Subsequently, seven experimental blocks were performed during intravenous administration of increasing levels of a single drug. For each block we set the drug concentration to a stationary level (remifentanil: 0.1, 0.15, 0.2, 0.25, 0.3, 0.35, 0.6 μg⋅kg^-1^⋅min^-1^ and propofol as target controlled infusion: 0.5, 1.0, 1.5, 2.0, 2.5, 3.0, 5.0 μg/ml). Placebo infusion rate was identical to remifentanil rate. During each stationary drug level participants were presented with approximately 1500 auditory clicks and their current level of sedation was quantified on a standardized sedation scale (Modified Observer’s Assessment of Alertness and Sedation Scale). Participants’ eyes were closed during the entire session.

Remifentanil (0.1, 0.15, 0.2, 0.25, 0.3, 0.35, 0.6 μg⋅kg^−1^⋅min^−1^) and propofol (target-controlled infusion of 0.5, 1.0, 1.5, 2.0, 2.5, 3.0, 5.0 μg/ml) were administered with a stepwise increase via an intravenous catheter (Graseby 3500, Graseby Medical Limited, United Kingdom). For target-controlled infusion of propofol, the Diprifusor system (Graseby 3500) was used. Up to the time of the study, there was no commercial target-controlled infusion system available for remifentanil. Placebo (0.9% NaCl) infusion rate corresponded to the one of remifentanil (0.05 mg/ml remifentanil) (Figure 1).

Each session consisted of a habituation period to familiarize the participant with the experimental procedure, a premedication period and up to seven treatment blocks of increasing drug concentration. If a participant became totally unresponsive to painful trapezius squeeze, the experimental session was stopped before the end of all seven treatment blocks. In addition, parameters such as heart rate, oxygen saturation and the mean arterial pressure were recorded to monitor the participant’s vital state.

After each change of infusion rate to the next higher level there was an equilibration period of 10 min. This 10-min interval allowed to obtain nearly steady-state conditions during recordings. During each stationary drug level AEP recordings were performed, followed by the quantification of the person’s vigilance, i.e., the current level of sedation. Other data obtained during the same experimental sessions (resting state, SSEP, iSEP) has been used in previous publications (Schmidt et al., 2007; Supp et al., 2011).

### Drug Characteristics

Remifentanil (*1-methyl-piperidine-4-carboxylate*) is a short-acting synthetic opioid analgesic agent, working as a specific μ-receptor agonist and, in addition to analgesia, causes a reduction in sympathetic nervous system tone and respiratory depression. The drug’s effects include a dose-dependent increase of muscle tone as well as a decrease in heart rate and arterial pressure, respiratory rate and tidal volume.

Propofol (*2,6-diisopropylphenol*) is an intravenous hypnotic agent widely used in standard clinical procedure to induce and maintain general anesthesia. Propofol acts as an agonist at the GABA-A receptor and causes reliable loss of consciousness. Discontinuation of drug administration results in rapid awakening and fast recovery from the clinical drug effects.

### Recordings and Stimulation

We recorded the electroencephalogram (EEG) of participants on an examination table with eyes closed. EEG was continuously recorded during the entire experiment from 126 sintered Ag/AgCl scalp-electrodes mounted in an equidistant cap layout (Falk Minow Services, Germany) referenced against the nose tip. Electrode impedances were kept below 10 kΩ before the start of the recordings. The data were recorded with an analog passband of 0.2–1,250 Hz and digitized at a sampling rate of 2,500 Hz using BrainAmp amplifiers (BrainProducts, Munich, Germany). Auditory stimuli were delivered as binaural clicks over a period of 3 min using a stimulation frequency of 9.1 Hz (∼1,500 stimuli), resulting in a trial length of 110 ms for each click.

### Processing

We performed all data processing in MATLAB (MathWorks) using custom scripts and the following open source toolboxes: BioSig^1^, EEGLAB^2^, and Fieldtrip^3^. For the analysis of mid-latency AEPs, the EEG data were band-pass filtered (2–250 Hz) and down-sampled to 500 Hz. In contrast, for the analysis of BAEPs, the EEG was band-pass filtered (100–1,000 Hz) and analyzed without down-sampling. Trials containing eye blinks, eye movements, muscle artifacts and signal drifts were rejected from further analysis based on both, semiautomatic procedures and visual inspection. In agreement with previous studies, we selected a centro-frontal electrode ROI (*n* = 7) to capture AEPs, consisting of the following electrodes (approximated locations): FCz, Fz, FCz-C2, FCz-C1, FCz-Fz, FCz-FC1, Fz-FC2, Fz-FC1 (Schwender et al., 1994; Bell et al., 2004; Haenggi et al., 2004).

### Auditory Evoked Potentials

Mid-latency AEPs were extracted from continuous EEG recordings by averaging EEG segments from 0 to 100 ms post stimulation onset.

#### Amplitude Complex

We extracted the temporal characteristics of AEPs by defining the local minima and maxima during pre-medication and each condition in the grand-average across all subjects. In particular, we obtained the time points of distinct mid-latency AEPs components (*Na*, *Pa*, and *Nb*) in a window 10–60 ms after stimulus onset and calculated the following compound measure of the amplitude complex as follows: *Pa* – (*Na* + *Nb*)/2 (Dutton et al., 1999).

#### 40 Hz Component

We obtained the frequency domain representation (spectral power) of mid-latency AEPs by applying a fast Fourier Transformation (FFT) on each trial’s 100 ms segment (0–100 ms after stimulus onset) in our predefined ROI, averaging across trials, and multiplying the complex Fourier-spectrum with its complex conjugate. As has been demonstrated before evoked power at 40 Hz can be used to investigate mid-latency AEPs (Galambos et al., 1981; Dutton et al., 1999; Plourde, 1999).

#### Brainstem Auditory Evoked Potentials

We analyzed BAEPs by calculating the root-mean square and averaging across the interval from 0 to 10 ms post stimulus onset, separately for each condition.

### Sedation Scale

At the end of each block, we quantified the participant’s vigilance using a standardized sedation scale, the Modified Observer’s Assessment of Alertness and Sedation Scale (MOAAS; Table 1). The rating of participant’s sedation level was always performed by the same investigator and ranged from MOAAS 5 (fully conscious) to MOAAS 1 (only responsive after painful physical stimulus) and MOAAS 0 (unresponsive) (Chernik et al., 1990; Schmidt et al., 2007; Supp et al., 2011). In addition, we recorded heart rate, oxygen saturation and mean arterial blood pressure to monitor the participants’ vital state.

**Table 1:** The Modified Observer’s Assessment of Alertness and Sedation Scale (MOAAS).

MOAAS score	Responsiveness criterion	Classification
5	Ready response to demand in normal tone	Conscious (CON)
4	Lethargic response to demand in normal tone	Reversible loss of consciousness (LOC)
3	Response only after loud and/or repeated demand	
2	Response only after mild prodding or shaking	
1	Response only after painful trapezius squeeze	
0	No response after painful trapezius squeeze	Unresponsive (UNR)

*Responsiveness scores of the Modified Observer’s Assessment of Alertness and Sedation Scale (Chernik et al., 1990; Schmidt et al., 2007). This standardized sedation scale was used to define participants’ state of vigilance.*

### Statistical Analysis

Statistical analyses were performed using R version 3.5.4^4^. First, we tested for significant drug-level effects on mid-latency AEPs and BAEPs. To account for missing values in higher drug levels we implemented linear mixed effects models (*lmer*, R-package: lmerTest, Version: 3.0-1) treating the eight drug levels (pre-medication and all seven drug blocks) as fixed effects and subjects (= 9) as random effects. We assessed changes of the mid-latency AEPs with drug-level by calculating a Pearson correlation over drug levels for each subject and, after Fisher transformation, by testing these correlation coefficients against zero across subjects with a two-sided *t*-test.

Second, we tested for drug-level effects on the sedation level (MOOAS). Taking into account the ordinal scale of MOAAS values, we followed an approach equivalent to the linear mixed effects model using a cumulative linked mixed model (*clmm*, R-package: ordinal).

Finally, we performed a linear mixed effects model analysis of the relationship between the amplitude of mid-latency AEPs and drug level and sedation level, respectively. We first tested for an interaction between drug level and sedation level. Neither in the remifentanil nor in the propofol condition we found significant interactions. Therefore, we established a linear mixed effects model without interaction and tested independent effects of drug level and sedation level (fixed effects), treating subjects as random effects (Cnaan et al., 1997). We restricted the fitting of the statistical models for each drug on the amplitude complex data in order to avoid problems associated with multiple comparison testing.

## Results

**FIGURE 2:**
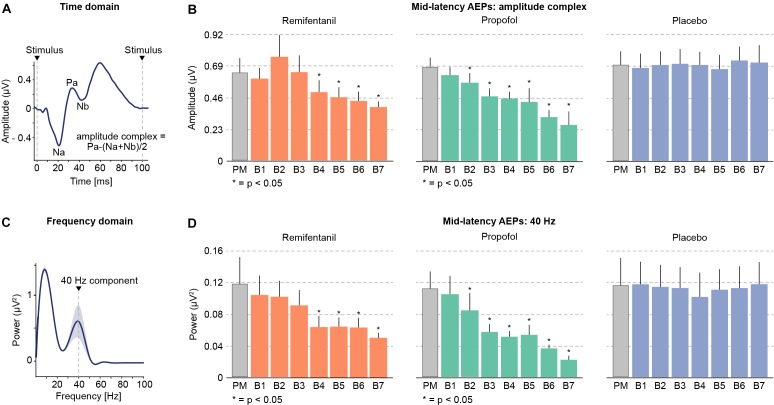
Mid-latency auditory evoked potentials (AEPs) during progressively increasing drug concentrations. **(A)** Time domain: the peaks of the most prominent mid-latency AEP components Na, Pa and Nb (10–50 ms after stimulus onset) were combined to compute an amplitude complex. **(B)** Increasing levels of remifentanil and propofol led to a profound reduction of mid-latency AEPs. **(C)** Frequency domain: A prominent peak in the 40 Hz frequency band reflects the mid-latency AEPs in the frequency domain. **(D)** The 40 Hz component also shows a prominent decrease with increasing levels of remifentanil and propofol. Placebo administration did not show any modulation of AEPs.

Mid-latency auditory evoked potentials can be investigated in the time and in the frequency domain (Galambos et al., 1981; Dutton et al., 1999; Plourde, 1999). We applied both approaches to assess if both approaches yield comparable results (Figure 2). In the time domain, we analyzed the size of an amplitude complex that combines the three most prominent mid-latency AEP components (*Na*, *Pa*, and *Nb*). In the frequency domain, we analyzed the AEPs power at 40 Hz, which well captures the mid-latency AEP components (Galambos et al., 1981; Dutton et al., 1999; Plourde, 1999).

We found that increasing dosages of both, remifentanil and propofol progressively suppressed mid-latency AEPs (Figure 2). In contrast, for placebo we did not find any significant changes during the entire experiment. We obtained very similar results for the amplitude complex (time domain) and for 40 Hz power (frequency domain), indicating the equivalence of both approaches. Specifically, we tested for significant drug effects using a linear mixed effects model with the main effect drug level and subjects as random effects term. This revealed the following results for each condition and analytical approach (40 Hz/amplitude complex): Remifentanil χ^2^(1) = 28.18, *p* < 0.0001/χ^2^(1) = 16.99, *p* < 0.0001; Propofol χ^2^(1) = 30.75, *p* < 0.0001/χ^2^(1) = 33.06, *p* < 0.0001; Placebo n.s. [χ^2^(1) = 0.12, *p* = 0.728]/n.s. [χ^2^(1) = 1.59, *p* = 0.212]. We further characterized the drug effect on mid-latency AEPs by calculating the correlation between drug level and AEP for each subject and by testing for a consistent correlation across subjects. This revealed a negative correlation of AEPs with increasing drug level for remifentanil and propofol for both analytical approaches. No correlation was found for placebo. Correlation coefficients for each condition and analytical approach (40 Hz/amplitude complex): Remifentanil, *r* = −0.70 (*p* = 0.0017)/*r* = −0.71 (*p* = 0.0014); Propofol, *r* = −0.85 (*p* < 0.0001)/*r* = −0.81 (*p* = 0.0025); Placebo, n.s. (*p* = 0.7102)/n.s. (*p* = 0.6336). Effect sizes of AEP decreases (calculated as percentage change for the maximum drug dosage relative to the premedication level) were (40 Hz/amplitude complex): Remifentanil, 58.07%/39.99%; Propofol, 80.65%/61.98%.

**FIGURE 3:**
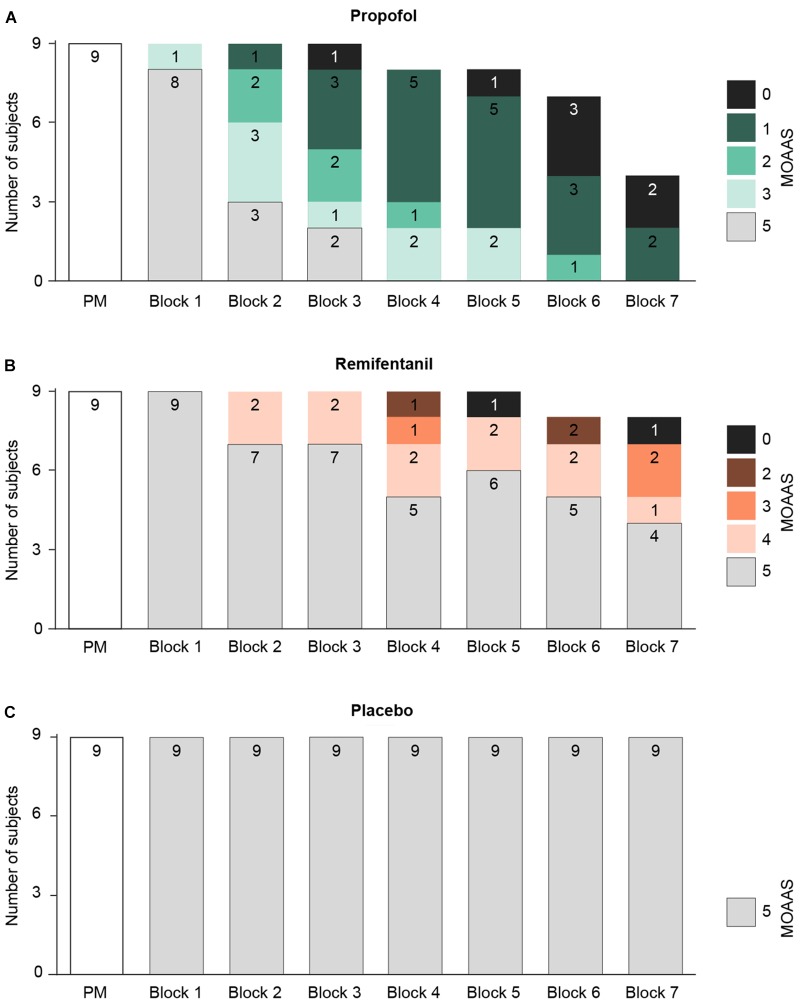
Changes of the participants’ vigilance were quantified using a standardized rating scale (Modified Observer’s Assessment of Alertness and Sedation Scale = MOAAS), ranging from MOAAS 5 (fully conscious) to MOAAS 1 (only responsive after a painful physical stimulus) and MOAAS 0 (unresponsive). Whenever a participant became totally unresponsive, the experiment was stopped before the end of all seven treatment blocks (block 1–7; PM, pre-medication block). **(A)** During progressive propofol medication the subjects’ vigilance was considerably diminished, reflected by a decrease in the MOAAS scale (MOAAS < 5). No participant received a rating of MOAAS 4. **(B)** Only high remifentanil concentrations increased the sedation level in about 50% of the participants. No participant received a rating of MOAAS 1. **(C)** For placebo, no changes in subjects’ sedation level were observed.

These results raise the question whether, as for hypnotic agents, the decline of mid-latency AEPs during remifentanil medication reflects the subjects’ sedation level, which may point to a common underlying mechanism. To address this, we quantified the behavioral effects of a given drug level by measuring the subjects’ vigilance, i.e., their sedation level, ranging from MOAAS 5 (“fully conscious”) to MOAAS 0 (“unresponsive”) (Figure 3). Linear mixed effects modeling revealed that the hypnotic drug propofol induced a profound and highly significant reduction of vigilance with increasing dosage [χ^2^(1) = 9.61, *p* = 0.0025]. In contrast, remifentanil induced a much weaker decrease of vigilance [χ^2^(1) = 6.04, *p* = 0.018]. While at highest propofol concentrations all subjects were sedated, at highest remifentanil concentrations, the sedation level increased in about half of the participants (Figure 3).

For hypnotic drugs, sedation has been shown to result in a reduction of mid-latency AEPs. To investigate whether the same pattern holds for an analgesic drug such as remifentanil, we analyzed both drugs separately in a linear mixed model. In particular, we sought to determine which factor (or combination of factors) is a significant predictor of the AEPs’ decline. To that end, we established linear mixed-models incorporating the factors drug level and sedation (Cnaan et al., 1997). This revealed diverging results for the different agents.

Importantly, the initial model included an interaction term, but did not reveal a significant interaction between drug level and sedation level. This was the case neither for propofol nor for remifentanil. Therefore, the subsequent models were established without interaction terms. We found that the decline of mid-latency AEPs during propofol administration was significantly predicted by the progressive sedation of subjects [χ^2^(2) = 39.94, *p* < 0.001], while at the same time AEP decline was not accounted for by the increase of propofol level (*p* = 0.395). In contrast, for the remifentanil condition, the linear mixed-model revealed a prominent effect of drug level on mid-latency AEPs [χ^2^(2) = 18.61, *p* < 0.0001], but no significant effect of the sedation level (*p* = 0.219).

**FIGURE 4:**
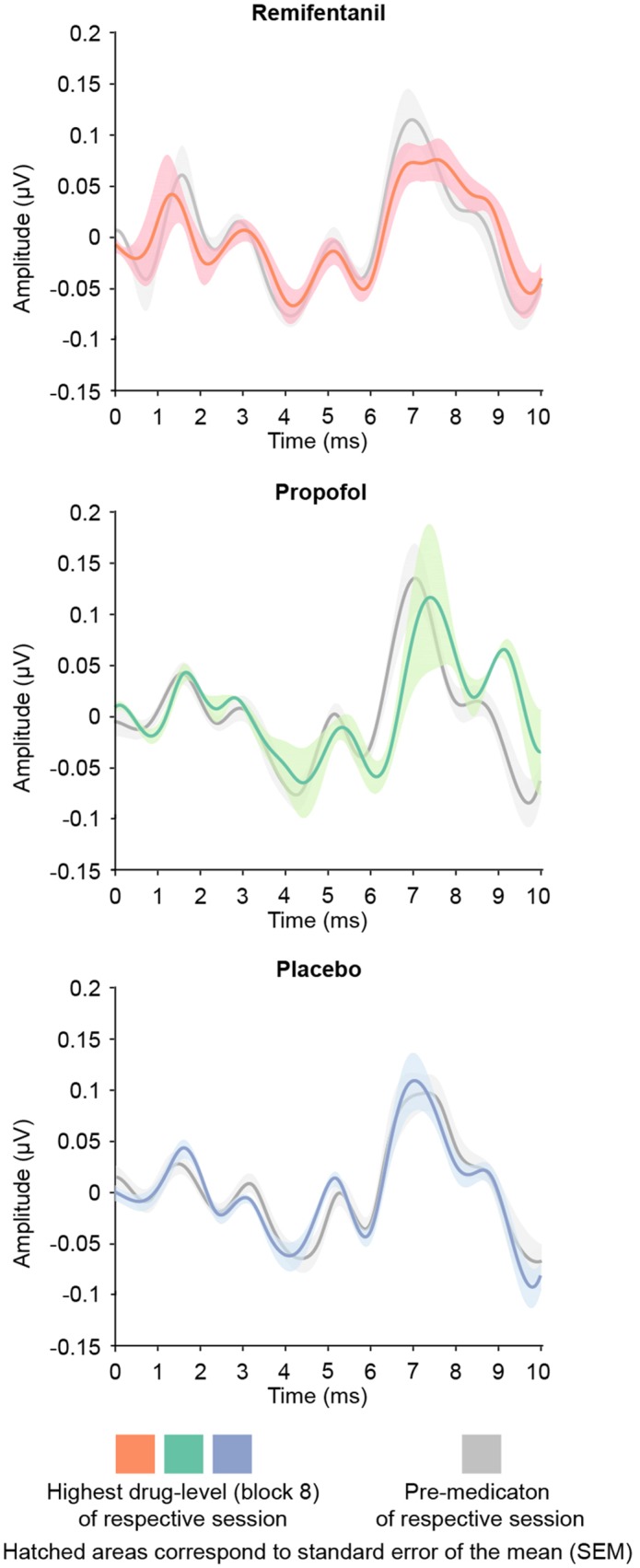
Brainstem auditory evoked potentials (BAEPs; 1–10 ms after stimulus onset) of the pre-medication block (gray) and the block of highest drug-level (colors). We quantified BAEPs by calculating the root-mean square and averaging across the entire interval 0–10 ms post stimulus onset. No drug showed a significant modulation of brainstem AEPs.

Unlike mid-latency AEPs, early brainstem auditory evoked potentials (BAEPs, 1–10 ms) were stable during increasing dosage of remifentanil and propofol (Figure 4). We assessed BAEPs by calculating the root-mean square and averaging across the entire 0–10 ms interval after stimulus onset, separately for each condition. A linear mixed effects model did not yield any significant BAEP modulation: Remifentanil, n.s. [χ^2^(1) = 0.35, *p* = 0.565]; Propofol, n.s. [χ^2^(1) = 2.68, *p* = 0.107]; Placebo, n.s. [χ^2^(1) = 0.117, *p* = 0.744].

## Discussion

As hypothesized, not only during propofol medication, but also during remifentanil medication mid-latency AEPs components decline in a dose-dependent manner. This held for the time and frequency domain representations of mid-latency AEPs, underlining the equivalence of both approaches.

Several previous studies did not find a modulating effect of opioids on mid-latency AEPs. Several reasons could account for these negative findings. One limitation of most studies that showed no effect is that they co-administered opioids and hypnotic agents (Crabb et al., 1996; Iselin-Chaves et al., 2000; Tooley et al., 2004; Wright et al., 2004; Schraag et al., 2006), which may decrease the reliability to detect an effect that can be specifically attributed to opioids. In contrast, for the present study the administration of only a single pharmaceutical agent at a time allowed to unequivocally attribute effects to the specific drug applied.

Another reason for previous negative and conflicting findings may be an insufficient drug dosage. As outlined above, Wright et al. (2004) did not find a direct effect of remifentanil on mid-latency AEPs, but concluded that this may reflect an insufficient dosage of remifentanil in their design. Indeed, they showed that the peak effect site concentration of remifentanil in their study was distinctly below the dosage used in another previous study by Crabb et al. (1996), in which a significant reduction of the *Pa*-amplitude during remifentanil infusion was found (12 ng/ml vs. 24.5 ng/ml). This suggests that also the negative (Iselin-Chaves et al., 2000; Schraag et al., 2006; Untergehrer et al., 2013) or non-significant (Schwender et al., 1993, 1995; Wright et al., 2004) findings of other studies may reflect the use of opioid dosages that were not sufficient to significantly depress mid-latency AEPs. In contrast, in the current study remifentanil concentrations were chosen high enough to produce an EEG effect (Shafer and Varvel, 1991; Egan et al., 1996).

Our linear mixed-model approach revealed an intriguing dissociation between remifentanil and propofol effects, supporting the initial hypotheses. During remifentanil dosage, the AEP decrease was associated with drug level increase, rather than with changes in sedation. In contrast, for propofol, the AEP decrease followed the progressive sedation of the subjects and was not accounted for by the increase of the drug. In other words, the decrease of mid-latency AEPs during propofol dosage reflects the individual functional consequence of medication, i.e., the sedation level, rather than merely the drug level. The opposite was the case for remifentanil.

Our findings indicate that different mechanisms may underlie the decline of mid-latency AEPs with the two drugs. For both, remifentanil and propofol, BAEPs were not modulated by drug dosage, i.e., neither of the two drugs affects the brainstem generators involved in the early auditory processing stages. This suggests that the effects of both, propofol and remifentanil on auditory evoked potentials must be attributed to higher brain regions.

Propofol causes a reliable sedation and seems to alter the general operating mode of the cerebral cortex (Supp et al., 2011). It is known to exert it’s major effect by binding as an agonist to gamma-aminobutric acid type A (GABA_A_) and as an antagonist to *N*-methyl-d-aspartate (NMDA) receptors in thalamo-cortical circuits (Brown et al., 2010). With increasing dosage, propofol enhances the GABA_A_-mediated inhibition of pyramidal neurons in the cortex and subcortical areas by interneurons, which synchronizes large populations of cortical neurons. On the neurophysiological level, this is reflected in a strong and large-scale coherent alpha-rhythm (Lopes da Silva, 1991; Huguenard and McCormick, 2007; Franks, 2008; Brown et al., 2010; Ching et al., 2010). This mechanism seems to inhibit the routing of sensory inputs through cortical networks and is associated with sedation (Supp et al., 2011; Chennu et al., 2016).

In contrast, remifentanil had little effect on the subjects’ sedation. The close correlation of the decrease in mid-latency AEPs with drug rather than with sedation level indicates that remifentanil blocks the sensory input in a way different from hypnotic agents. Remifentanil is a specific μ-receptor agonist. It’s target receptors are located in the spinal cord, the periaqueductal gray, the medulla oblongata, the thalamus and the limbic system (Schwender et al., 1993; Trescot et al., 2008; Brown et al., 2010). Importantly, no opioid receptors were reported in primary auditory cortex (Wright et al., 2004). Opioids are assumed to mediate some of their effects, e.g., analgesia, by inhibiting the afferent input to the cortex (Schwender et al., 1993). Indeed, it has been shown that morphine blocks subcortical somatosensory evoked potentials (SSEP) at the thalamic level (Abdulla and Aneja, 1993).

Our findings support the idea that opioids such as remifentanil block sensory signal transmission by binding to specific receptors in the thalamus, which does not affect BAEPs or sedation level, but results in a decline of the cortical mid-latency AEPs (Crabb et al., 1996). In line with the well-known thalamic relay functions (McCormick and Bal, 1994), one could therefore speculate, that μ-receptors in the thalamus act as well as a specific gating mechanism for sensory information (Freye et al., 1986; Abdulla and Aneja, 1993).

In conclusion, our results shed light to the question whether or not opioids such as remifentanil affect mid-latency AEPs.

Our results suggest that in a clinical setting where hypnotic drugs are used in combination with opioids such as remifentanil a monitored decrease in mid-latency AEPs might not unequivocally be attributed to the hypnotic drug. Furthermore, we found that AEP decreases that were associated with robust sedation for propofol, were not associated with robust sedation for remifentanil (e.g., compare remifentanil block 6 and propofol block 5 in Figures 2, 3). Thus, one could speculate that the interpretation of a decrease in mid-latency AEPs caused by remifentanil based on the predicted effect of propofol could potentially lead to a mis-classification of the patient’s sedation level (Meyer and Mendelson, 1961; Daunderer and Schwender, 2004; Salomons et al., 2004; Sebel et al., 2004; American Society of Anesthesiologists Task Force on Intraoperative Awareness, 2006).

However, some limitations should be recognized. The ramped up infusion rates of remifentanil used in the present study reflect a comparatively high dose range that might not often be used in clinical anesthesia. Therefore, in clinical settings the changes in mid-latency AEPS induced by remifentanil might be smaller than in the present study. Additionally, the effects of remifentanil alone on mid-latency AEPs in unstimulated volunteers may be different compared to its contribution during clinical anesthesia with varying levels of stimulation and in combination with propofol. Further studies that specifically investigate propofol-remifentanil interactions in the presence or absence of stimulation are required to assert this.

## Author Contributions

FH and GS analyzed the data and drafted the manuscript. JH was involved in data analysis and revision of the manuscript. AE was involved in developing the experimental idea and revision of the manuscript. MS was involved in developing the experimental idea, data analysis and revision of the manuscript.

## Conflict of Interest Statement

JH is a full-time employee of F. Hoffmann-La Roche Ltd. The remaining authors declare that the research was conducted in the absence of any commercial or financial relationships that could be construed as a potential conflict of interest.

## Footnotes

1 http://www.biosig.sourceforge.net/

2 http://www.sccn.ucsd.edu/eeglab/

3 http://www.ru.nl/fcdonders/fieldtrip/

4 http://www.r-project.org/
